# Transcriptomic Analysis Reveals the Response of the Bacterium *Priestia Aryabhattai* SK1-7 to Interactions and Dissolution with Potassium Feldspar

**DOI:** 10.1128/aem.02034-22

**Published:** 2023-05-08

**Authors:** Hui Yang, Lanxiang Lu, Yifan Chen, Jianren Ye

**Affiliations:** a College of Forestry and Co-Innovation Center for Sustainable Forestry in Southern China, Jiangsu Key Laboratory for Prevention and Management of Invasive Species, Nanjing Forestry University, Nanjing, Jiangsu, China; Colorado School of Mines

**Keywords:** mineral weathering, transcriptome, *Priestia aryabhattai*, reactive oxygen species, pyruvate, malic enzyme

## Abstract

Potassium feldspar (K_2_O·Al_2_O_3_·6SiO_2_) is considered to be the most important source of potash fertilizer. The use of microorganisms to dissolve potassium feldspar is a low-cost and environmentally friendly method. Priestia aryabhattai SK1-7 is a strain with a strong ability to dissolve potassium feldspar; it showed a faster pH drop and produced more acid in the medium with potassium feldspar as the insoluble potassium source than in the medium with K_2_HPO_4_ as the soluble potassium source. We speculated whether the cause of acid production was related to one or more stresses, such as mineral-induced generation of reactive oxygen species (ROS), the presence of aluminum in potassium feldspar, and cell membrane damage due to friction between SK1-7 and potassium feldspar, and analyzed it by transcriptome. The results revealed that the expression of the genes related to pyruvate metabolism, the two-component system, DNA repair, and oxidative stress pathways in strain SK1-7 was significantly upregulated in potassium feldspar medium. The subsequent validation experiments revealed that ROS were the stress faced by strain SK1-7 when interacting with potassium feldspar and led to a decrease in the total fatty acid content of SK1-7. In the face of ROS stress, strain SK1-7 upregulated the expression of the *maeA-1* gene, allowing malic enzyme (ME2) to produce more pyruvate to be secreted outside the cell using malate as a substrate. Pyruvate is both a scavenger of external ROS and a gas pedal of dissolved potassium feldspar.

**IMPORTANCE** Mineral-microbe interactions play important roles in the biogeochemical cycling of elements. Manipulating mineral-microbe interactions and optimizing the consequences of such interactions can be used to benefit society. It is necessary to explore the black hole of the mechanism of interaction between the two. In this study, it is revealed that *P. aryabhattai* SK1-7 faces mineral-induced ROS stress by upregulating a series of antioxidant genes as a passive defense, while overexpression of malic enzyme (ME2) secretes pyruvate to scavenge ROS as well as to increase feldspar dissolution, releasing K, Al, and Si into the medium. Our research provides a theoretical basis for improving the ability of microorganisms to weather minerals through genetic manipulation in the future.

## INTRODUCTION

Potassium is the seventh most abundant element in the Earth's crust, and soils hold large reserves of potassium (1). Potassium ions (K^+^) play an important role in promoting plant growth; over 60 enzymes require K^+^ for activation, and they play a crucial role in a number of fundamental processes, including enzyme activation, membrane transport, anion neutralization, etc. (2, 3). K^+^ deficiency in plants leads to yellowing or even necrosis of leaves, sensitivity to environmental stress, slow growth, poor fruit quality, and low yield (4, 5). Unfortunately, K^+^ is present mainly in insoluble form in abundant silicate minerals, and in this form, it cannot be directly absorbed and used by plants (6).

Potassium feldspar is a sedimentary rock and silicate mineral that is rich in potassium. At present, the industrial methods used to produce potash from potash ore can be broadly divided into the following three types: direct crushing, high-temperature calcination, and wet chemical methods (7, 8). These methods are simple and efficient; however, they involve lengthy processes, require large investments, have high costs, increase tailing production, exhibit low economic efficiency, and cause environmental pollution, thus hindering further development of this industry (9). In recent years, weathering (dissolution and release) of minerals through microorganisms has been performed in many countries (10–12); this method can greatly reduce environmental pollution (6). Additionally, this method facilitates the supply of nutrients to the microorganisms themselves, as well as the acquisition of enzymatic metal cofactors (13, 14). However, the efficiency of microbial potassium release needs to be improved compared to that of physical and chemical methods.

To improve the efficiency of microbial weathering of minerals, the mechanism of microbial-mineral interactions needs to be investigated; this research will facilitate modification of microorganisms at the molecular level to improve the efficiency of ore weathering. According to recent research, the mechanism of microbial weathering of minerals mainly includes the following processes: acidolysis (as a result of the attachment of protons to organic acid molecules, the pH of the solution decreases, and cations such as iron, potassium, and magnesium are released into the solution [15, 16]); chelation (cations might dissolve faster when chelating molecules form strong bonds with them or with mineral surfaces [17]); and oxidoreduction (mineral surfaces, such as those of silicates, are the sites of complex oxidoreduction reactions that require direct contact between bacteria and mineral surfaces [18, 19]). Acid digestion is the main mechanism for the weathering of potassium-bearing minerals (20–23). Chen et al. (24) showed that *Rhizobium* H66 promotes potassium feldspar weathering via glucose metabolism. Wang et al. (25) showed that Pseudomonas azotoformans produces more gluconic acid in the process of weathering black mica, having a strong effect. This bacterial strain also has a well-developed acid tolerance system, which allows it to release acidic substances and weather minerals while resisting acid stress itself.

It is possible that microorganisms can rapidly release large amounts of acidic substances within a short period of time after encountering minerals, causing a rapid decrease in the pH of the surrounding environment because they are under environmental stress. A number of metal-bearing minerals can produce reactive oxygen species (ROS) by abiotically dissolving metal ions, participating in redox reactions and forming surface defects (26). The term ROS encompasses oxygen free radicals, such as superoxide anion radical (O2^·−^) and hydroxyl radical (^·^OH), and nonradical oxidants, such as hydrogen peroxide (H_2_O_2_) and singlet oxygen (^1^O_2_) (27). He et al. (28) found that the quartz surface can produce sufficient H_2_O_2_ and O_2_ under oxygen-free conditions. Excess ROS can lead to bacterial DNA damage and lipid and protein oxidation and pose a serious threat to the bacterium itself (29–31). Therefore, acid production by microorganisms under stress may be used to resist the damage caused by ROS. Conversely, in the process of weathering minerals, microorganisms can accidentally release metals, including Cs, Al, Hg, Pb, Cu, Zn, Co, and Mn, and these heavy metals have severe effects on the microbial community microorganisms themselves (32). The hard structures on the surface of minerals can cause damage to bacterial cell membranes during cultivation in the presence of minerals under laboratory conditions, leading to protein leakage and placing the bacteria under growth stress. In summary, few studies have examined whether microorganisms are subjected to stress and whether acid production during microbial weathering of minerals is a defense response caused by stress, so it is worthwhile to further investigate these aspects.

In our laboratory, Bacillus aryabhattai SK1-7 was isolated and screened from poplar inter-root soil with a strong ability to promote tomato growth (33). This bacterium can effectively dissolve insoluble potassium and release soluble potassium ions, with a release rate of 32.6%. The pH of SK1-7 under incubation with an insoluble potassium source decreases rapidly in a relatively short period of time, with the strain mainly secreting malic acid, formic acid, acetic acid, and citric acid, thereby releasing potassium, silicon, and aluminum ions from potassium feldspar (34). Gupta et al. (35) divided *Bacillus* species into 17 different branches by phylogenomic and comparative genomic analyses, and *B. aryabhattai* was renamed Priestia aryabhattai. In our study, short-term mineral weathering experiments were conducted on *P. aryabhattai* SK1-7, and the main factors causing acid production in SK1-7 were analyzed. Comparative transcriptomic analysis was performed to identify the differentially expressed genes (DEGs) of strain SK1-7 under culture conditions with potassium feldspar as the insoluble potassium source versus K_2_HPO_4_ as the soluble potassium source, and subsequent annotation of the DEGs was conducted against the Gene Ontology (GO) and KEGG pathway databases. Key metabolic pathways involved in acid production from SK1-7-weathered minerals and the bacterium’s own stress resistance and repair pathways were predicted and validated in an attempt to reveal the relationship between the defense responses of SK1-7 and K^+^ release under stress and to provide new insights into microbe-ore interactions.

## RESULTS

### Gene ontology enrichment analysis of differentially expressed genes.

The analyses showed that all sequencing data were of high quality (Percentage of the bases with Qphred > 30 [Q30] ≥ 92%) (Table 1; see also Fig. S1 in the supplemental material). The number of clean reads (passing-filter reads) in each group varied from 16.03 Mb to 16.31 Mb, with GC content between 40.39 and 40.74% (Table 1).

**TABLE 1:** Summary of the sequencing data of SK1-7

Sample	Raw reads (M)	Raw bases (G)	Clean reads (M)	Clean bases (G)	Valid bases (%)	Q30^*a*^ (%)	GC (%)
SK1_7_1	16.58	2.49	16.22	2.37	95.23	92.61	40.74
SK1_7_2	16.54	2.48	16.20	2.37	95.40	92.77	40.61
SK1_7_3	16.39	2.46	16.03	2.34	95.21	92.54	40.39
SK1_7_4	16.55	2.48	16.30	2.39	96.43	93.79	40.48
SK1_7_5	16.55	2.48	16.31	2.39	96.41	93.91	40.43
SK1_7_6	16.46	2.47	16.22	2.38	96.29	93.90	40.64

a Percentage of the bases with Qphred > 30 (error rate < 0.1%).

**FIG 1:**
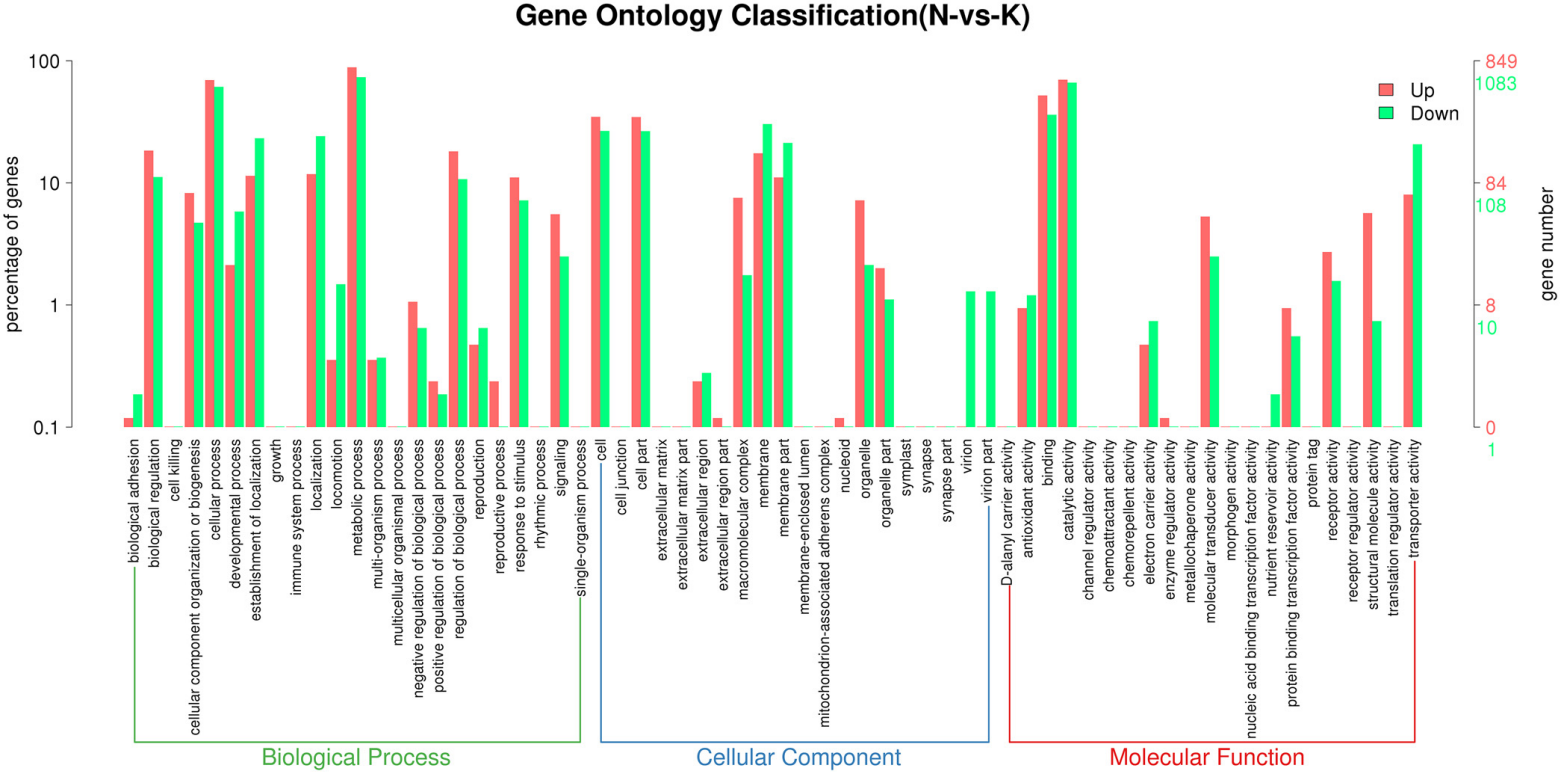
GO annotation analysis of DEGs from SK1-7 grown in FM (feldspar) medium (N in the figure) compared to FM (K_2_HPO_4_) medium. SK1-7 grown in FM (feldspar) medium (K in the figure). Red indicates GO level 2 entries with upregulated DEG enrichment, green indicates GO level 2 entries with downregulated DEG enrichment, the horizontal axis shows the entry name, and the vertical axis indicates the number of genes in the corresponding entry and their percentages.

The number of downregulated genes (1,859) was more than 1.5 times higher than that of upregulated genes (1,223), suggesting that potassium feldspar inhibits gene expression in SK1-7 to some extent (see Fig. S2 in the supplemental material). GO functional annotation of the DEGs showed that 4,565 genes, accounting for 48.47% of the total, were involved in biological processes (BPs); 2,197 (23.33%) DEGs were associated with cellular components (CCs); and 2,656 (28.20%) genes were involved in molecular functions (MFs). In the BP category, the DEGs were involved in the regulation of metabolic, cellular, and biological processes; in the CC category, most of the DEGs were associated with cells, membranes, and macromolecular complexes; and in the MF category, the catalytic activity, molecular transduction activity, and transport protein activity pathways were activated (Fig. 1).

GO enrichment analysis showed that metabolic process (GO:0008152) and cellular process (GO:0009987) were the two most enriched subgroups in the BP category, while cell (GO:0005623) and membrane (GO:0016020) were the two most enriched subgroups in the CC category, and catalytic activity (GO:0003824) and binding (GO:0005488) were the two most enriched subgroups in the MF category (see Fig. S3 in the supplemental material). The results imply that potassium feldspar could significantly affect the physiological functions and metabolism of SK1-7.

### KEGG enrichment analyses of DEGs.

**FIG 2:**
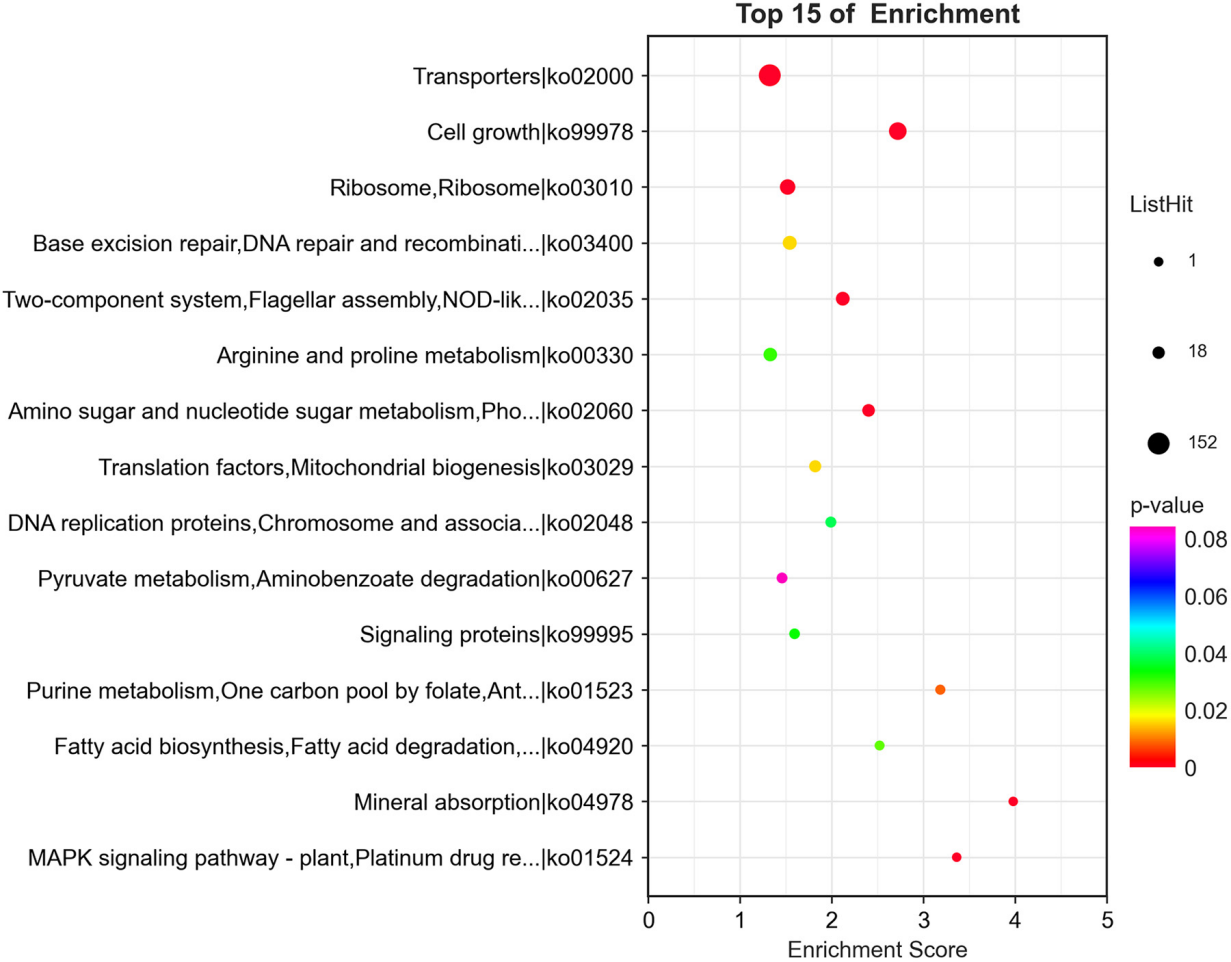
KEGG enrichment analysis of the top 15 DEGs (screening of pathway entries corresponding to the number of DEGs greater than 2, sorted by the −log_10_ *P* value corresponding to each entry from largest to smallest) shown as a bubble plot.

KEGG pathway enrichment analyses demonstrated that the DEGs could be divided into 249 pathways involved in various functions, including basic metabolism, signaling, and transport. To characterize the effects of potassium feldspar on the metabolic pathways of strain SK1-7, we performed a clustering analysis of DEGs based on the 15 KEGG pathways in this study (Fig. 2). These pathways included pyruvate metabolism (27 DEGs, 11 upregulated and 16 downregulated), DNA repair (7 DEGs, 5 upregulated and 2 downregulated), ribosome (44 DEGs, 43 upregulated and 1 downregulated), cell growth (13 DEGs, 7 upregulated and 6 downregulated), two-component system (77 DEGs, 46 upregulated and 31 downregulated), transport (85 DEGs, 30 upregulated and 55 downregulated), and arginine and proline metabolism (42 DEGs, 13 upregulated and 29 downregulated).

### Transcriptomic analysis results for DEGs in strain SK1-7.

Differential expression of representative genes was examined in the pyruvate metabolism, DNA repair, two-component system, and resistance to oxidative stress pathways, which were the main pathways enriched by DEGs during the interaction of potassium feldspar with strain SK1-7 (Table 2). The upregulated genes associated with oxidative stress included *catB*, *trxA*, *butE*, and *ahpC*. The upregulated enzymes associated with DNA repair were NTH, UNG, and MPG. Among the DEGs associated with pyruvate metabolism, the upregulated genes included *maeA-1* and *fumA* and *fumB* (the genes encoding fumarate hydratase, catalyzing the production of malate from fumaric acid), and the downregulated genes included *mqo* and *mdh*, encoding malate dehydrogenase, which catalyzes the production of oxaloacetate from malate. KinE, KapB, and Spo0F are associated with a two-component system involved in synthesizing biofilm-associated proteins in response to external environmental changes. Upregulation of the expression of proteins related to malate utilization, including MalK, MalR, MaeA, and MaeN, was observed.

**TABLE 2:** Genes upregulated in the presence of potassium feldspar grouped by metabolic pathway based on KEGG pathway enrichment analysis

Pathway and gene name	Orf_name	Function or description of product	Fold change (log_2_)
Pyruvate metabolism			
*fumA*	chr_336	Fumarate hydratase	1.86
*maeA-1*	chr_2048	Malate dehydrogenase	2.35
*korB*	chr_4166	2-Oxoglutarate	2.13
DNA repair			
NTH	chr_1335	Endonuclease III	1.85
UNG	chr_5232	Uracil-DNA glycosylase	1.19
MPG	chr_2144	DNA-3-methyladenine glycosylase	2.21
Two-component system			
*envZ*	chr_1626	Osmolarity sensor histidine kinase	2.66
*kdpD*	chr_3112	Sensor histidine kinase	3.57
*kdpC*	chr_3113	Potassium-transporting ATPase	3.38
*kdpB*	chr_3114	Potassium-transporting ATPase	3.53
*kdpA*	chr_3115	Potassium-transporting ATPase	3.86
Oxidative stress			
*catB*	chr_5265	Catalase	3.42
*trxA*	chr_4778	Thioredoxin 1	1.54
*btuE*	chr_1972	Glutathione peroxidase	1.61
*ahpC*	chr_918	Peroxiredoxin	5.17

### RT-qPCR was used to validate the transcriptome results.

**FIG 3:**
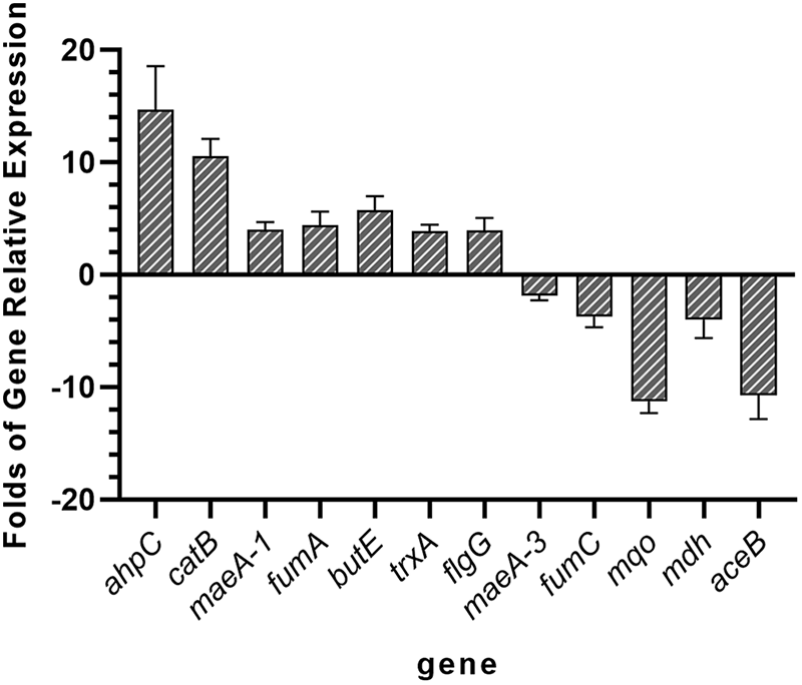
qPCR verification of the DEGs of SK1-7 identified by transcriptome sequencing. The *gyrA* gene was used as an internal control.

A total of 12 DEGs involved in pyruvate metabolism and oxidative stress pathways in SK1-7 were examined by reverse transcriptase quantitative PCR (RT-qPCR). The RT-qPCR results showed that the expression of the *ahpC*, *catB*, *maeA-1*, *fumA*, *butE*, *trxA*, and *flgG* genes was upregulated and that of the *maeA-3*, *fumC*, *mqo*, *mdh*, and *aceB* was downregulated in strain SK1-7 grown in fermentation medium (FM) (feldspar) medium compared to FM (K_2_HPO_4_) medium. This is in general agreement with the transcriptomic results (Table 3 and Fig. 3).

**TABLE 3:** Differentially expressed genes of interest selected from the transcriptomic data for qPCR verification

Gene (orf_name)	Function or description of product	*P* value	Fold change
*ahpC* (chr_918)	Alkyl hydroperoxide reductase	2.27E−84	36.06
*catB* (chr_5265)	Catalase	8.19E−44	10.74
*maeA-1* (chr_2048)	Malate dehydrogenase	6.59E−26	5.10
*fumA* (chr_336)	Fumarate hydratase	2.13E−16	3.64
*butE* (chr_1972)	Glutathione peroxidase	3.31E−16	3.04
*trxA* (chr_4778)	Thioredoxin	2.87E−13	2.89
*flgG* (chr_5162)	Flagellar basal-body rod protein	2.68E−08	3.60
*maeA-3* (chr_2685)	Malate dehydrogenase	7.24E−23	−25.64
*fumC* (chr_2371)	Fumarate hydratase	2.60E−09	−2.86
*mqo* (chr_2935)	Malate dehydrogenase	3.58Ev22	−5.00
*mdh* (chr_4818)	Malate dehydrogenase	0.00031	−1.72
*aceB* (chr_2950)	Malate synthase	9.19E−16	−2.94

### Changes in pH.

**FIG 4:**
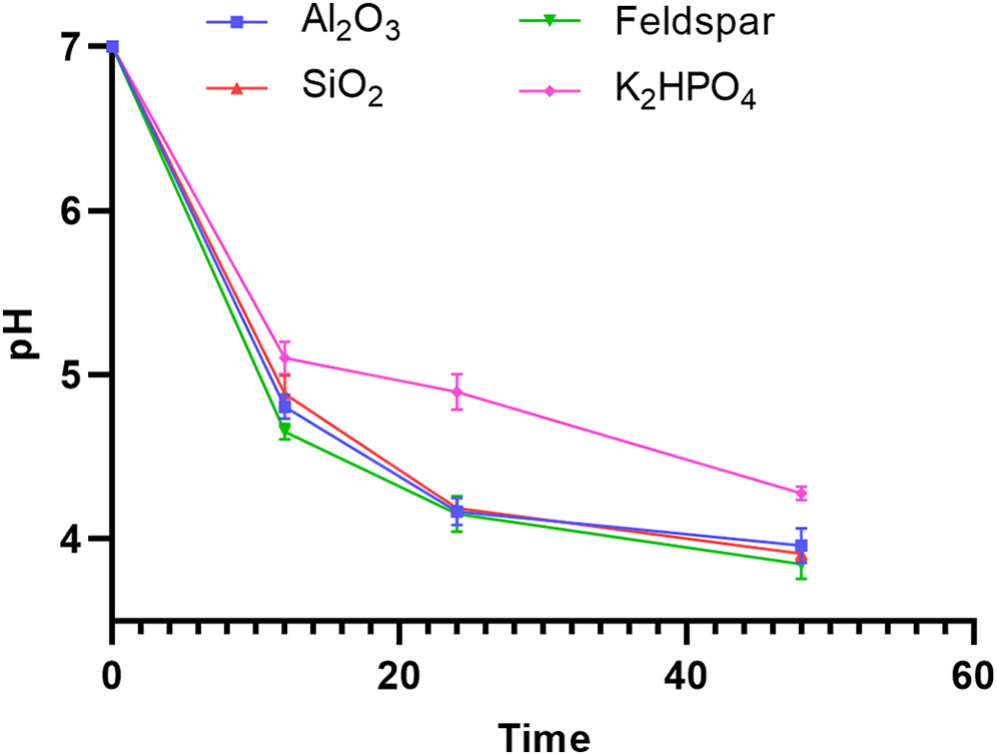
The change in pH of strain SK1-7 in FM (feldspar), FM (SiO_2_), FM (Al_2_O_3_), and FM (K_2_HPO_4_) media during the first 48 h (*n* = 3).

SK1-7 cells were grown in the control medium, namely, FM (K_2_HPO_4_), and in the experimental group media, namely, FM (feldspar), FM (SiO_2_), and FM (Al_2_O_3_). In the first 12 h, the pH of the experimental group dropped rapidly from the initial value of 7.0 to approximately 4.7, and that of the control group dropped from 7.0 to approximately 5.1; from 12 to 24 h, the pH of the experimental group dropped from 4.7 to approximately 4.0 and that of the control group dropped from 5.1 to approximately 4.9; after 24 h, the pH of the experimental group dropped to 3.8 and then tended to remain almost unchanged, while that of the control group remained unchanged at approximately 4.3 (Fig. 4). The difference among the experimental groups was not significant (*P *> 0.05), while the difference between the experimental and control groups was significant (*P *< 0.0001). According to the results, the addition of ore could rapidly lower the environmental pH in the growth medium of strain SK1-7, eventually causing it to remain more acidic than the control.

### SK1-7 inner and outer membrane damage.

**FIG 5:**
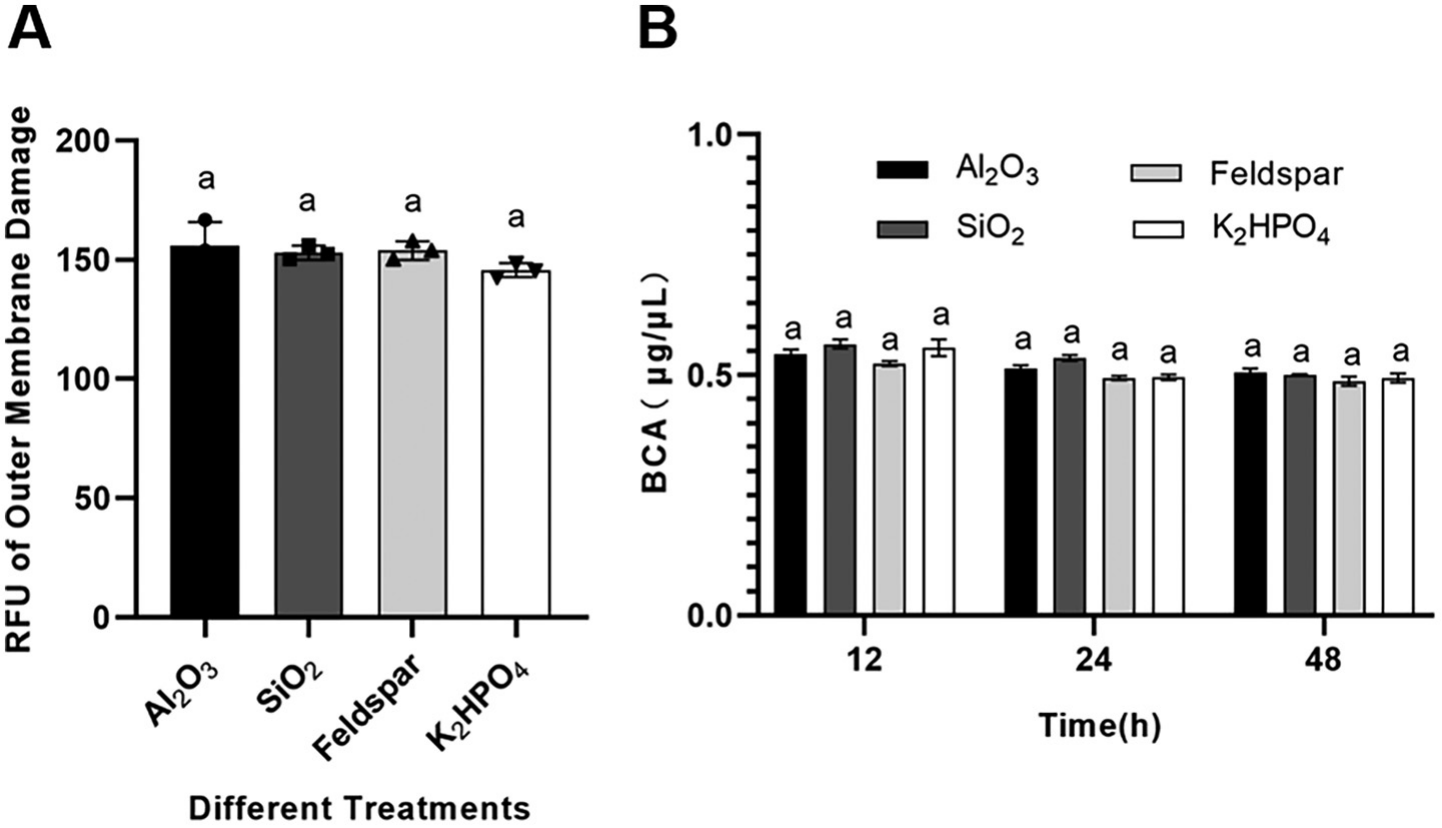
Effect of the ore on the degree of damage to the inner and outer membranes of SK1-7. (A) Relative fluorescence units (RFUs) of SK1-7 in FM (Al_2_O_3_), FM (SiO_2_), FM (feldspar), and FM (K_2_HPO_4_) media, corresponding to outer membrane damage. (B) Protein content in the supernatants of SK1-7 cells at 12 h, 24 h, and 48 h in the above four media (*n* = 3). The same letter indicates that the difference is not significant (*P* > 0.05).

The mean relative fluorescence units measured for strain SK1-7 grown in FM (Al_2_O_3_), FM (SiO_2_), FM (feldspar), and FM (K_2_HPO_4_) media for up to 12 h were 156.1 ± 9.69, 152.9 ± 2.94, 153.8 ± 3.90, and 145.7 ± 2.95, respectively, with no significant differences among the groups (*P *> 0.05) (Fig. 5A). This result indicates that the outer membrane of strain SK1-7 was not significantly damaged during the interaction with the mineral. The protein content in the culture supernatant was generally maintained at 0.5 μg/μL according to the 12, 24, and 48 h measurements, with no significant difference among the groups (*P *> 0.05) (Fig. 5B). This result indicates that there was no protein leakage from SK1-7 and that the inner cell membrane was not damaged. Overall, both the inner and outer membranes of SK1-7 remained intact and were not mechanophysically damaged by minerals.

**FIG 6:**
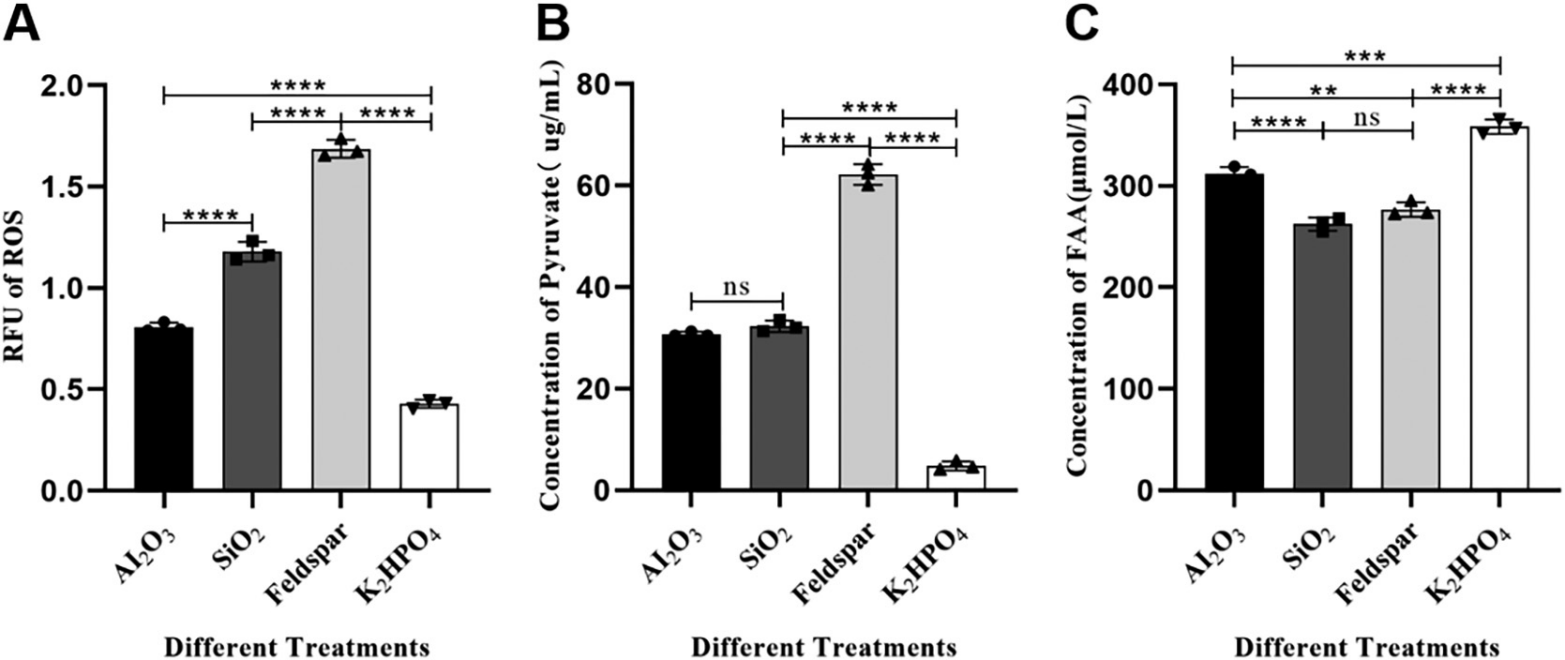
Effect of potassium feldspar and each of these oxides on ROS stress and substance metabolism in SK1-7 cells. (A) SK1-7 grown in these four media until the 8th hour of intracellular ROS content. (B) The amount of pyruvate secreted by the bacterium SK1-7 grown in these four media for 12 h. (C) Total fatty acid content produced by SK1-7 grown in these four media for 12 h (*n* = 3). **, *P* < 0.01; ***, *P* < 0.001; ****, *P* < 0.0001.

Determination of ROS content. The ROS levels in SK1-7 at the early stage of growth (at 8 h) in the four media were as follows: the relative ROS content in FM (feldspar) medium was 1.69 ± 0.04, which was 4 times higher than that in the control medium FM (K_2_HPO_4_), 2 times higher than that in FM (Al_2_O_3_) medium, and 1.4 times higher than that in FM (SiO_2_) medium, and the relative ROS content in the control medium FM (K_2_HPO_4_) was 0.43 ± 0.02, which was 25%, 36%, and 53% that in the FM (feldspar), FM (SiO_2_), and FM (Al_2_O_3_) media, respectively. Significant differences were observed among the groups (*P* < 0.0001) (Fig. 6A). This result indicates that the addition of minerals led to the accumulation of ROS in SK1-7, and the degree of accumulation showed a tendency to increase with the amount of ore substances added to the medium.

### Differences in the production of pyruvate secreted by SK1-7 in different media.

The amounts of exocytosed pyruvate from strain SK1-7 grown in the above four media for up to 12 h were as follows (Fig. 6B): among the experimental groups, the amount of pyruvate secreted by SK1-7 was 62.21 ± 2.05 μg/mL in FM (feldspar) medium, 32.31 ± 1.11 μg/mL in FM (SiO_2_) medium, and 30.75 ± 0.45 μg/mL in FM (Al_2_O_3_) medium. In contrast, in the control group, the amount of pyruvate secreted in FM (K_2_HPO_4_) medium was 4.78 ± 0.90 μg/mL. The difference in the amounts of propionic acid secreted by SK1-7 in FM (SiO_2_) and FM (Al_2_O_3_) media was not significant (*P* > 0.05), but the difference compared to the amount secreted in FM (feldspar) medium was significant (*P* < 0.0001), and the amount of the latter was approximately twice as much as that of the former. The differences between each experimental group and the control group were significant (*P* < 0.0001). According to the results of this study, the amount of pyruvate secreted by strain SK1-7 was positively correlated with the amount of minerals added and the amount of ROS.

### Differences in the yield of fatty acids synthesized by SK1-7 in different media.

The total fatty acid levels in strain SK1-7 at 12 h of growth in different media were as follows (Fig. 6C): the amounts of fatty acids produced by SK1-7 in FM (feldspar), FM (SiO_2_), and FM (Al_2_O_3_) media were 276.7 ± 7.23 μmol/L, 262.3 ± 6.66 μmol/L, and 312.0 ± 6.56 μmol/L, respectively, in the experimental groups. The amount of fatty acids produced by SK1-7 in FM (K_2_HPO_4_) medium in the control group was 358.3 ± 7.10 μmol/L. The differences between FM (feldspar) and FM (SiO_2_) media were not significant (*P* > 0.05), while those between FM (SiO_2_) and FM (Al_2_O_3_) media were significant (*P* < 0.0001) as were those between each experimental group and the control group (*P* < 0.0001). Thus, the amount of fatty acids produced by SK1-7 was significantly higher in the control group than in the experimental group, suggesting that exocytosis of pyruvate may have depleted the substrate for fatty acid synthesis by SK1-7 or that the fatty acids were oxidized by ROS.

### RT-qPCR analysis of genes related to the pyruvate metabolism pathway.

**FIG 7:**
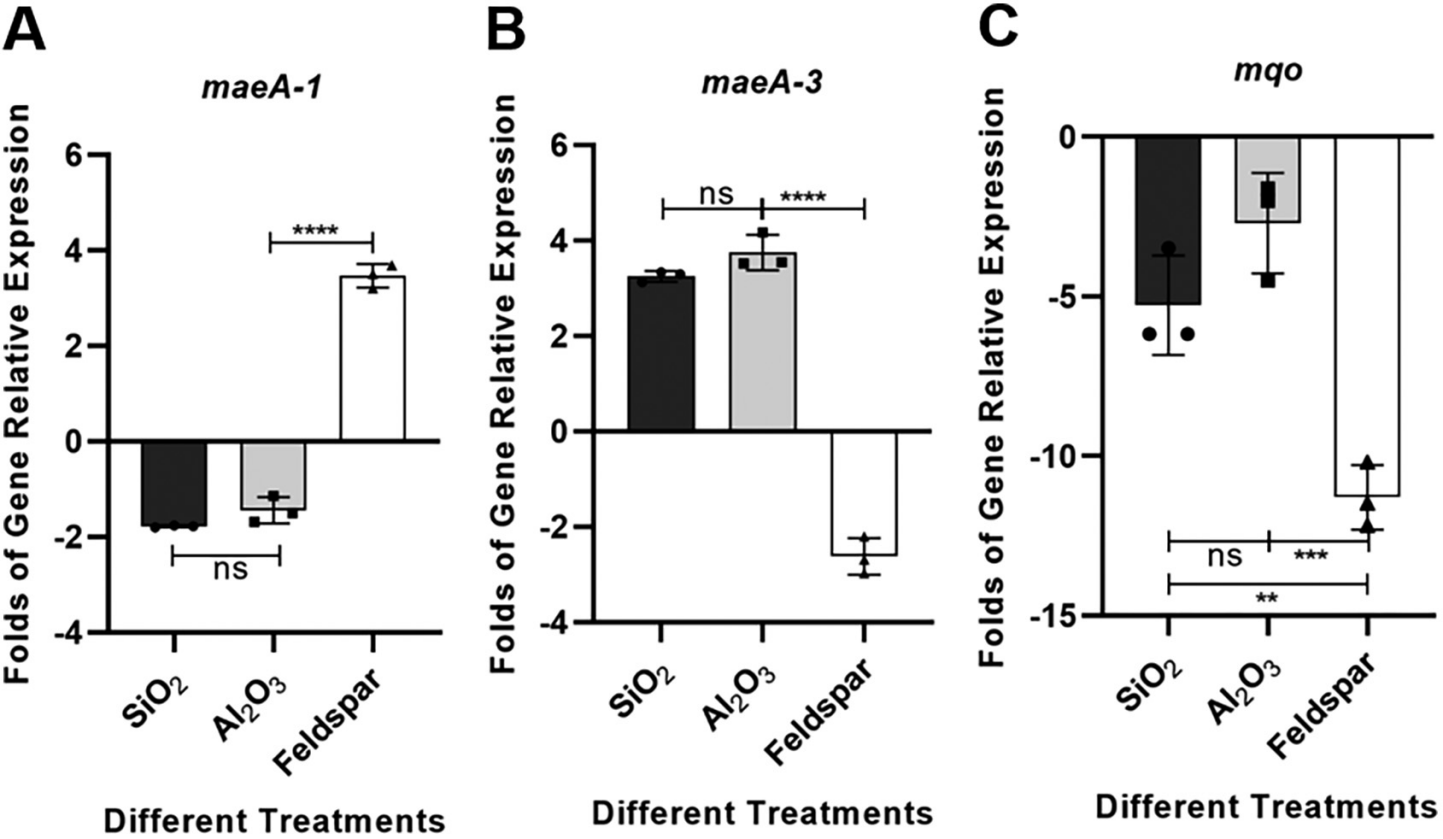
Expression of the *maeA-1*, *maeA-3*, and *mqo* genes in SK1-7 grown in FM (SiO_2_), FM (Al_2_O_3_), FM (feldspar), and FM (K_2_HPO_4_). **, *P* < 0.01; ***, *P* < 0.001; ****, *P* < 0.0001.

The transcriptomic results of SK1-7 in the experimental medium FM (feldspar) and control medium FM (K_2_HPO_4_) showed 5.1 times higher *maeA-1* gene expression, 3.9% higher *maeA-3* expression, and 19.5% higher *mqo* expression than that in the control group. To investigate whether potassium feldspar and the different types of oxides contained in it (mainly SiO_2_ and Al_2_O_3_) led to the differences in the expression of these three genes, RT-qPCR validation was performed using SK1-7 grown in FM (K_2_HPO_4_) medium as a control. The level of *maeA-1* gene expression in FM (SiO_2_) medium was 56.2% that in the control, FM (Al_2_O_3_) medium was 70.9% that in the control, and FM (feldspar) medium was 3.13-fold that in the control (Fig. 7A). For the *maeA-3* gene, SK1-7 was expressed 3.25 times more in FM (SiO_2_) medium than in the control, 3.75 times more in FM (Al_2_O_3_) medium, and 38.7% more in FM (feldspar) medium than in the control (Fig. 7B). Across the different treatment groups, *mqo* expression was downregulated, with the expression level in FM (SiO_2_) medium being 20.3% that in the control, that in FM (Al_2_O_3_) medium being 44.8% that in the control, and that in FM (SiO_2_) medium being 8.9% that in the control (Fig. 7C). This result suggests that *maeA-1* and *maeA-3* may have different kinds of metal active sites.

## DISCUSSION

Our results differ from those of previous studies that have explored the mechanisms of microbial potassium solubilization and the transformation of minerals as a microbial metabolic response. Environments containing minerals can have harmful effects on microorganisms.

In this study, the early response of strain SK1-7 to interaction with potassium feldspar was explored by transcriptomic analysis. The possible types of stress imposed by potassium feldspar on SK1-7 and the series of self-repair and defense mechanisms adopted by the strain to respond to this stress were analyzed. Potassium feldspar and various oxides were found to cause no cell membrane damage to strain SK1-7 (Fig. 5A and B), and the possibility of membrane damage during the interaction of strain SK1-7 with potassium feldspar was ruled out.

Aluminum is present in potassium feldspar and is toxic to microorganisms (21, 26). Interestingly, in our study, we found that strain SK1-7 grew in FM (Al_2_O_3_) and FM (SiO_2_) media with the same pH variations (Fig. 4). Previous studies in our laboratory showed that the aluminum and silica ion levels in the fermentation filtrate of strain SK1-7 in FM (feldspar) showed a gentle increasing trend for the first 7 days (34). It is assumed that the gradual shift to acidic conditions in the medium resulted in the release of Al^3+^, Si^4+^, etc., from potassium feldspar. SK1-7 may avoid the DNA damage caused by heavy metal poisoning through osmotic barrier efflux, active efflux, and inhibition of influx (36). It is presumed that the stress it faces is not caused by specific heavy metal (aluminum) ions.

The accumulation of ROS in SK1-7 showed an increasing trend with mineral supplementation in FM (Al_2_O_3_), FM (SiO_2_), and FM (feldspar) (Fig. 6A). While the total fatty acid content of SK1-7 decreased with the increase in added mineral content, all of the values were significantly lower than that of the control group (Fig. 6C). The decrease in fatty acid content rules out the possibility that SK1-7 uses fatty acids to repair the membrane (Fig. 5A and B), so fatty acids may be subject to oxidation by ROS, or the source of synthetic fatty acids may be reduced due to exocytosis of pyruvate. This indicates that SK1-7 was indeed subjected to oxidative stress due to ROS accumulation. Some studies have shown that through Fe(III) reduction and transformation, interactions between microbial communities and Fe minerals in soil slurries synergistically drive HO^·^ production (37). Notably, in this study, trace amounts of FeCl_3_ were added to each medium, with the exact amount differing among experimental groups because potassium feldspar itself contains 1.08% Fe_2_O_3_. It is speculated that the accumulation of ROS may be associated with minerals, Fe^3+^, and microorganisms, and whether the redox reaction involving Fe plays a decisive role in the interaction between microorganisms and minerals is worthy of further investigation. Endogenous basal levels of bacterial ROS are necessary to sustain life (38).

**FIG 8:**
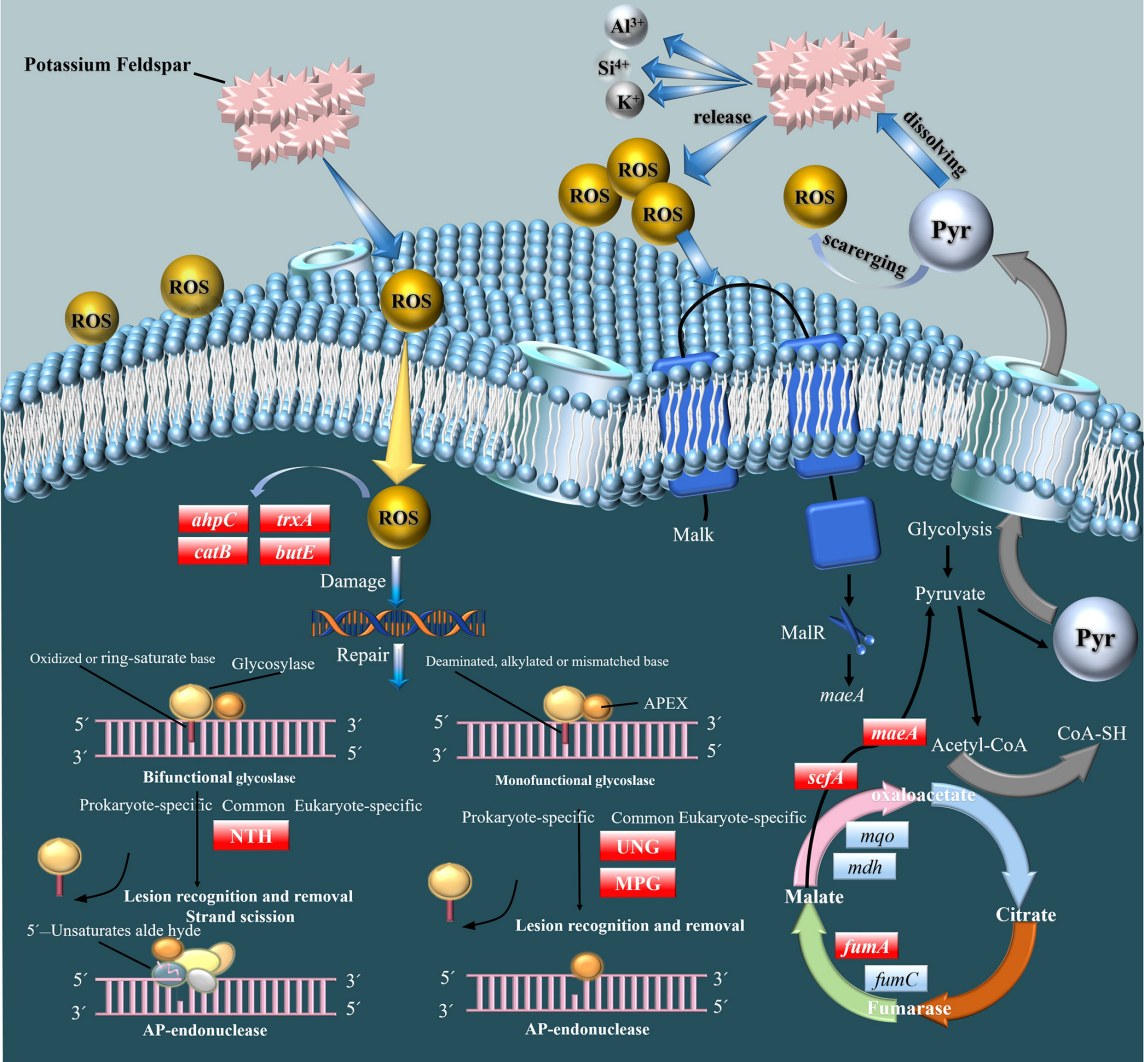
Patterns of relevant metabolic responses and differential gene expression in the transcriptome. Red boxes represent upregulated genes or proteins, and blue boxes represent downregulated genes or proteins, *ahpC* (encoding peroxiredoxin), *butE* (encoding glutathione peroxidase), *trxA* (encoding thioredoxin), *catB* (encoding catalase), *maeAs* (encoding malic enzyme 2), *mqo* (encoding malate dehydrogenase); NTH (endonuclease III), UNG (uracil-DNA glycosylase), MPG (DNA-3-methyladenine glycosylase); MalK (a multiple sugar transport system ATP-binding protein), MalR (a response regulator MalR).

ROS cause the upregulation of genes related to oxidative stress in SK1-7 cells, including *catB* and *ahpC*. These enzymes play a direct role in scavenging peroxides, breaking down hydrogen peroxide to molecular oxygen and water, thereby performing a detoxification function and protecting cells from the toxic effects of hydrogen peroxide (17, 39). The gene *butE* encodes glutathione peroxidase, which catalyzes the conversion of GSH to GSSG and the reduction of toxic peroxides to nontoxic hydroxy peroxides (29). *trxA* encodes a thioredoxin-reducing protein that acts as a hydrogen donor in many reduction reactions, regulating and stabilizing the intracellular redox potential (39) (Table 2). Although minerals provide these enzymes with metal cofactors, microbial activity is influenced both negatively and positively by minerals, which thus act as a double-edged sword (32). Interestingly, in antimicrobial material development, metal nanoclusters achieve bacteriostatic effects by interacting with Gram-negative bacterial cells, leading to the accumulation of ROS (40). In another study, it was found that SiO_2_ nanoparticles caused more damage to Gram-negative bacteria than to Gram-positive bacteria, which was closely related to the thick peptidoglycan layer on the cell membrane of Gram-positive bacteria (29), which could explain the accumulation of ROS that caused stress but not lethality to the Gram-positive bacterium SK1-7 in our study and the ability of the organism to adopt relevant defensive measures. Furthermore, mineral ROS production may be an important selection pressure driving microbial evolution, and *P. aryabhattai* SK1-7 performs well under these conditions, exhibiting the ability to repair damaged DNA via base excision repair and resist oxidative stress (Fig. 8).

ME2, encoded by the *maeA* gene, plays an important role in active resistance to ROS stress in *P. aryabhattai* SK1-7 (Fig. 6 and 8). Previous studies in our laboratory showed that SK1-7 secreted more malic acid in FM (feldspar) than in a control (34). In the study by Zhang et al. (41), malate metabolism in Bacillus licheniformis was regulated by MalK/MalR in the presence of malic acid. When MalK receives the extracellular malate signal, communication between MalK and MalR occurs via phosphoryl group transfer (42). Ganesh et al. (43) showed that malic acid secretion enhances *maeA* gene expression, and malic acid also specifically induces transcription of the *maeA* gene (44). This was one of the factors associated with the upregulation of *maeA* gene expression in the present study.

Regarding the function of malic enzyme (ME2), in plants, the response to intracellular and extracellular oxidative stress leads to the accumulation of NADP-ME2 transcripts (45). Overexpression of nicotinamide adenine dinucleotide phosphate-malic enzyme (DADP-ME) in the cytosol of *Arabidopsis* and rice enhances osmotic and salt stress tolerance (46). The role of malic enzyme in plant protection as well as resistance to external stresses has been widely reported, while its role in microbes is less well reported; we speculate that ME2 may be important in the resistance of SK1-7 to ROS stress. Furthermore, the malic enzymes encoded by the *maeA-1* and *maeA-3* genes may be regulated by different metal ions at different metal active sites, such as K^+^ and AI^3+^ and Si^4+^. In future research, we will analyze the enzymatic properties of these two proteins and determine whether they perform different biological functions.

In the present study, the amount of pyruvate secreted by SK1-7 showed a positive correlation with the ROS level in the bacterium (Fig. 6A and B). Zhang et al. (47) found that pyruvate is a stress molecule in *S. aeruginosa*, and oxidative and osmotic/salt stresses lead to the accumulation of pyruvate, which scavenges stress-induced ROS and promotes fungal growth; thus, stress-induced pyruvate accumulation is a cross-protective mechanism. Other similar results have been observed; in a study by Kawasaki and Kamagata (48), pyruvate, a well-known scavenger of H_2_O_2_, could effectively scavenge H_2_O_2_ when added to the culture medium; thus, it seems that the pyruvate produced by SK1-7 can effectively purge the ROS induced by minerals, providing protection to the bacterium itself. At the same time, pyruvic acid has a direct dissolution effect on potassium feldspar, thus releasing a large amount of K^+^, AI^3+^, and Si^4+^ (49). To further investigate whether pyruvate scavenges ROS in SK1-7, we will construct strains overexpressing ME2 in the future, which will allow us to explore whether SK1-7 could accelerate mineral solubilization via increased pyruvate secretion.

Our study provides new insights into the causes and mechanisms underlying acid production by microorganisms during their interactions with minerals. Mineral-induced production of ROS causes upregulation of the expression of a series of antioxidant genes in SK1-7. Excess ROS leads to DNA damage, causing upregulated expression of a series of DNA repair genes. This is a passive method of self-defense and repair. In the active defense approach, the SK1-7 two-component system senses changes in the external environment and induces upregulated expression of the downstream malic enzyme (ME2) encoded by *maeA* genes. Sucrose in the medium undergoes glycolysis to glucose via the tricarboxylic acid (TCA) cycle and produces malate, which in turn produces more pyruvate in response to ME2. Pyruvate is then secreted from the cell. Pyruvate acts as both a scavenger of external ROS and an accelerator of potassium feldspar dissolution to release K^+^, Al^3+^, Si^4+^, etc. (Fig. 8). It better explains the early responses to these two interactions at the molecular level.

However, there are also certain shortcomings and directions that need to be further explored in depth in future research. The use of potassium feldspar to study microbial potassium solubilization is a classic research method. A question raised by the novel insights from this study is that if microbial dissolution of minerals is a defensive response, can the effects of functional microorganisms on minerals (potassium feldspar, mica stone, etc.) in a laboratory setting occur in a soil environment? It is worthwhile to continue in-depth research and exploration on this subject.

## MATERIALS AND METHODS

### Bacterial strain and minerals.

*P. aryabhattai* SK1-7 was isolated from the rhizosphere of *Populus alba* L. and preserved at the Laboratory of Forest Pathology, Nanjing Forestry University. The potassium feldspar used as the silicate mineral in this study was sieved to collect grains (74 μm) and cleaned as described by Sheng et al. (15). The composition of the potassium feldspar was as follows: 61.06% SiO_2_, 15.45% Al_2_O_3_, 1.08% Fe_2_O_3_, 0.33% MgO, 0.23% CaCl_2_, 8.45% K_2_O, and 2.43% Na_2_O, with 2.72% loss on ignition.

### Short-term mineral weathering experiment.

Single colonies of SK1-7 were inoculated in LB medium, which had the following formulation: tryptone, 10.0 g; yeast extract, 5.0 g; NaCl, 10.0 g; double-distilled water (ddH_2_O), 1,000 mL; pH 7.2 to 7.4. The bacteria were incubated at 30°C and 200 rpm for 12 h for growth to logarithmic phase. The cells were collected by centrifugation, washed, and then resuspended in sterile distilled water to a final concentration of 10^8^ cells mL^−1^. Then, the cells were inoculated (1% inoculation volume) in fermentation medium with different potassium sources and cultured at 30°C and 200 rpm for 12 h. The fermentation medium (hereafter FM) was formulated as follows: 10.0 g of sucrose, 1 g of Na_2_HPO_4_, 1 g of MgSO_4_·7H_2_O, 0.0005 g of FeCl_3_, 0.5 g of (NH4)_2_SO_4_, 0.2 g of yeast extract, and 1,000 mL of ddH_2_O, pH 7.0 to 7.2. In the experimental group, 12 g of potassium feldspar powder was added to each 1 L of FM, and the medium was recorded as FM (feldspar). In the control group, the potassium feldspar powder was replaced with 0.1% K_2_HPO_4_, and the other components of the medium were kept unchanged; this medium was recorded as FM (K_2_HPO_4_).

### Testing the effect of different elements in potassium feldspar on SK1-7.

The percentage of each oxide in potassium feldspar was used as a reference, i.e., 12 g of potassium feldspar powder was replaced with 7.33 g of SiO_2_ and 1.854 g of Al_2_O_3_ per 1 L of fermentation medium, while the other components were kept constant; the two media were recorded as FM (SiO_2_) and FM (Al_2_O_3_), respectively. The pH values of the media were measured at 12, 24, and 48 h for the four different treatment groups.

### Transcriptome sequencing.

SK1-7 grown in FM (feldspar), as the experimental group, or in FM (K_2_HPO_4_), as the control group, was incubated at 30°C and 200 rpm for 12 h. Insoluble material in the medium was removed through a 1-μm aqueous filter membrane, and the organisms were collected by centrifugation at 8,000 rpm for 3 min.

SK1_7_1, SK1_7_2, and SK1_7_3 were the control groups, wherein SK1-7 was cultured in FM (K_2_HPO_4_) medium; SK1_7_4, SK1_7_5 and SK1_7_6 were the experimental groups, wherein SK1-7 was cultured in FM (feldspar) medium (Table 1).

The above samples were subjected to transcriptome sequencing (Shanghai OE Biotech Co., Ltd.). Total RNA was extracted using the mirVana microRNA (miRNA) isolation kit (Ambion) following the manufacturer’s protocol. RNA integrity was evaluated using the Agilent 2100 Bioanalyzer (Agilent Technologies, Santa Clara, CA, USA). Samples with an RNA integrity number (RIN) of ≥7 were subjected to further analysis. The libraries were constructed using TruSeq Stranded Total RNA with Ribo-Zero Gold according to the manufacturer’s instructions. Then, these libraries were sequenced on the Illumina sequencing platform (HiSeq 2500 or another platform), and 150 bp/125 bp paired-end reads were generated. We used the known genome sequence of SK1-7 (NCBI accession number PRJNA716807) as a reference, and the expression abundance of each gene in each sample was identified by sequence similarity comparison. Rockhopper 2 was used to determine the number of reads aligned to the gene in each sample and then to calculate the reads per kilobase per million (RPKM) value (50). The estimateSizeFactors function of the R package DESeq (2012) was used to standardize counts, and the nbinomTest function was used to calculate the *P* value and fold change of the difference. Differentially expressed transcripts with *P* values less than 0.05 and a fold change greater than 2 were selected, and GO and KEGG enrichment analyses of the DEGs were performed by hypergeometric distribution tests to determine the biological functions or pathways that were mainly affected by the DEGs.

### Real-time quantitative PCR.

The bacteriophage was prepared using the same method as that used for the transcriptome. Then, a total RNA extraction kit (Accurate Biology Co., Ltd., Changsha) was used to extract RNA from the SK1-7 strain. Using the protocol for the RNA reference reverse transcription kit (Accurate Biology Co., Ltd., Changsha), 1 μg of total RNA was used for PCR in a thermocycler (Eppendorf no. 5345/015458; Germany). All qPCR primers were designed using the online tool Primer 3 (https://bioinfo.ut.ee/primer3/) (Table 4). The *gyrA* gene was used as an internal reference. Three biological replicates and three technical replicates were performed for each experimental sample. The 2^−ΔΔ^*^CT^* method was used to analyze the PCR data (21).

**TABLE 4:** Primers used for qPCR analysis in this study

Primer	Sequence (5′-3′)
ahpC F	GCTCTATCAGATCACTACGA
ahpC R	GCTACGCCTTCTTCTTCA
catB F	CATTAGAAGACGCTGATACG
catB R	CGGACGGTTAATCGGTAG
maeA-1 F	AGCAGGAATCGACCCAAGC
maeA-1 R	CCAGTGTAACAGTGCATTAGGGA
fumA F	CGTGATTTAGACGGCATTCG
fumA R	TCGCCACCGATTCCAAC
butE F	CGTGCTGTTGATTGTGAATA
butE R	TTGAACTTCCTGGCTCTTG
trxA F	GCTATTGCACATGCTACAG
trxA R	TTATCGTTAAGTTCGCCATC
flgG F	TACGGGCGTTGACCAGTTT
flgG R	TCTACGCCTGCTGTTTCTCG
maeA-3 F	GCTTCCTATTATTTACACGCCTAC
maeA-3 R	TATCTTCGCTGTCGAGTCCTAA
fumC F	CTACACCGCTAACACTAGGACAA
fumC R	GCTGCTGATTTCTTTCGCTAC
mqo F	CAGACGGCTCGTGGGAAGTA
mqo R	ACCGGGAACCCTCCAATAT
mdh F	TTGAAAGAACACGCAAAGGC
mdh R	AGGTAAGCAATCGCTGGTAAA
aceB F	TTGGCGTAATGGACGAAGA
aceB R	TTGGAATCCGCTTGCTAATC
gyrA F	AGAAGTAGACAAGGGTGAGTGGG
gyrA R	AACGGATGACTTTGACACCTTG

### Measurement of SK1-7 outer membrane damage.

SK1-7 cells were incubated in FM (feldspar), FM (SiO_2_), FM (Al_2_O_3_), and FM (K_2_HPO_4_) media for 12 h. Each culture was diluted to an optical density at 600 nm (OD_600_) of 0.50. Then, 1 mL was transferred to a new 1.5-mL centrifuge tube, and the sample was centrifuged at 13,000 rpm for 1 min to collect the bacterial pellet. A fluorescence assay kit was used for detection of bacterial outer membrane damage by the 1-*N*-phenylnaphthylamine uptake method (GenMed, Scientifics, Inc. USA). The bacteria were washed with GenMed buffer, suspended in the buffer, and mixed thoroughly. Then, the bacterial culture was pipetted into the sample wells of a 96-well plate at 190 μL per well, and 5 μL of GenMed staining solution was added to each sample well. The plate was then incubated for 5 min at 25°C. The plate was immediately placed in a microplate fluorometer (Fluoroskan Ascent FL; Thermo, USA). The relative fluorescence unit (RFU) value was obtained at an excitation wavelength of 355 nm and an emission wavelength of 460 nm to compare the degree of cell membrane damage of strain SK1-7 among different media.

### Measurement of SK1-7 inner membrane damage.

The SK1-7 strain was cultured in the above four media for 12 h, 24 h, and 48 h, and the cultures were then centrifuged at 13,000 rpm for 3 min. To assess bacterial intracellular membrane damage, a bicinchoninic acid (BCA) protein quantification kit (Vazyme Biotech Co., Ltd.) was used to measure protein leakage. The protein standard curve was prepared with 0, 1, 2, 4, 8, 12, 16, and 20 μg of standard solution; 20 μL of the supernatant of the sample to be tested was added to 200 μL of BCA working solution, shaken, and mixed, and the mixture was then placed at 37°C for 20 to 30 min. Absorbance values were measured at A562 nm with a microplate reader (Multiskan Spectrum; Thermo, USA), and absorbance values of samples without BSA were used as a blank control. The standard curve was plotted with the protein content (μg) as the horizontal coordinate and the absorbance value as the vertical coordinate. The protein content of the samples was calculated by using the standard curve based on the measured absorbance values. The result was used to determine the damage to the inner membrane of SK1-7 cells.

### Determination of ROS content in SK1-7 cells.

The relative ROS content was measured using an ROS assay kit (Biosharp, Hefei, China) with the fluorescent probe H2DCFH-DA. SK1-7 cells were incubated in the above four media for 8 h, recovered and adjusted to 1 × 10^8^ cells mL^−1^. Subsequently, the probe was loaded in the bacterium, the cells were incubated for 30 min at 37°C in an incubator protected from light, and fluorescence was measured with a microplate fluorescence reader (Fluoroskan Ascent FL; Thermo, USA) at an excitation wavelength of 488 nm and emission wavelength of 525 nm.

### Measurement of pyruvate content in the bacterial culture supernatants.

Strain SK1-7 was cultured in the above four media for 12 h and centrifuged at 13,000 rpm for 3 min. The supernatant was collected and pipetted into 1 mL of extract. Then, the sample was placed in an ice box for 30 min and centrifuged at 8,000 × *g* for 10 min at room temperature. The supernatant was taken for testing. A pyruvate content assay kit (Beijing Solarbio Science & Technology Co., Ltd.) was used, wherein the standards were diluted with distilled water to 25, 12.5, 6.25, 3.125, 1.5625, 0.78125, 0.39, and 0 μg/mL. Then, 300 μL of standard or sample was added to 100 μL of reagent I in a 1.5-mL EP tube, mixed well, and allowed to stand for 2 min. Subsequently, 500 μL of reagent II was added, and the sample was mixed well. The absorbance value at 520 nm was then measured using a UV-visible spectrophotometer (Lambda 365; PerkinElmer, USA). Zeroing was performed with distilled water. A standard curve was established based on the concentration of the standards and the measured values; for this curve, *x* is the sodium pyruvate content (μg/mL), and *y* is the absorbance value. The measured absorbance values were plugged into the standard curve equation to calculate the pyruvate content in the supernatants of the above four media for SK1-7 growth.

### Determination of total FAA content.

SK1-7 cells were incubated in the above four media for 12 h, and the culture was then adjusted to 1 × 10^8^ cells mL^−1^. According to the protocol for an fatty acid amine (FAA) content kit (Beijing Solarbio Science & Technology Co., Ltd.), 1 mL of extraction solution was added to the culture, followed by ultrasonic crushing (ice bath; power, 200 W; ultrasonication, 3 s; interval, 10 s; repeat 30 times). Then, the sample was allowed to stand for 30 min, centrifuged at 8,000 × *g*, and allowed to stand at room temperature for 10 min; the supernatant was then used for analysis. The standards were diluted with chloroform to 0.8, 0.6, 0.4, 0.2, 0.1, 0.05, and 0.025 μmol/mL. Then, 50 μL of the sample to be measured or the standard was pipetted out, and 500 μL of reagent I and 200 μL of reagent II were added. The sample was then shaken thoroughly for 10 min and centrifuged at 3,000 rpm for 10 min. Subsequently, 200 μL of the supernatant was added to 800 μL of reagent III, shaken thoroughly for 2 min and allowed to stand for 15 min. The absorbance values were measured at 550 nm by a microplate reader (Multiskan Spectrum; Thermo, USA). A standard curve was prepared to calculate the total FAA content in SK1-7 cells in the four different media.

### Statistical analysis.

One-way analysis of variance (ANOVA) (Duncan’s test) was performed using SPSS; different letters indicate significant differences (*P* < 0.05).

### Data availability.

Raw transcriptome data of the six samples were deposited in the SRA database (Sequence Read Archive, NCBI) with accession numbers SRR22520263, SRR22520262, SRR22520261, SRR22520260, SRR22520259, and SRR22520258.
