# Supplementary material for: Transcriptomic Analysis Reveals the Response of the Bacterium Priestia Aryabhattai SK1-7 to Interactions and Dissolution with Potassium Feldspar
Source: Appl Environ Microbiol. 2023 May 8;89(5):e02034-22. doi: 10.1128/aem.02034-22 (PMC10231193; doi:10.1128/aem.02034-22)

Fig. S1. Quality control of the sequencing results for the SK1-7 transcriptomes. (a) Quality score across all bases (Sanger/Illumina 1.9 sequencing). (b) Sequencing saturation curve. (c) Gene coverage analysis.

(a)

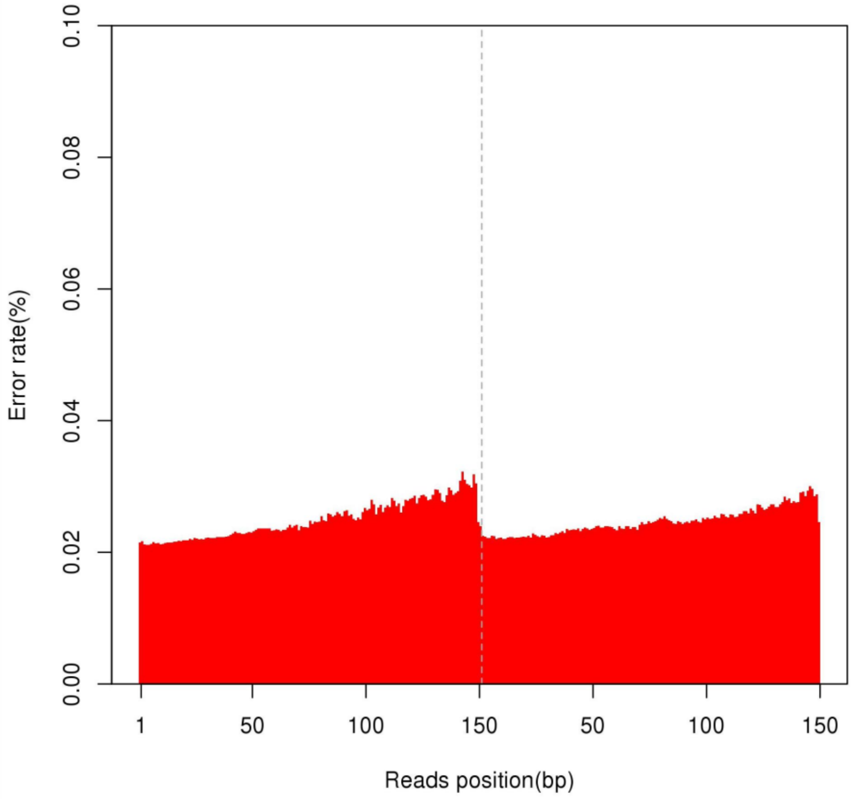

(b)

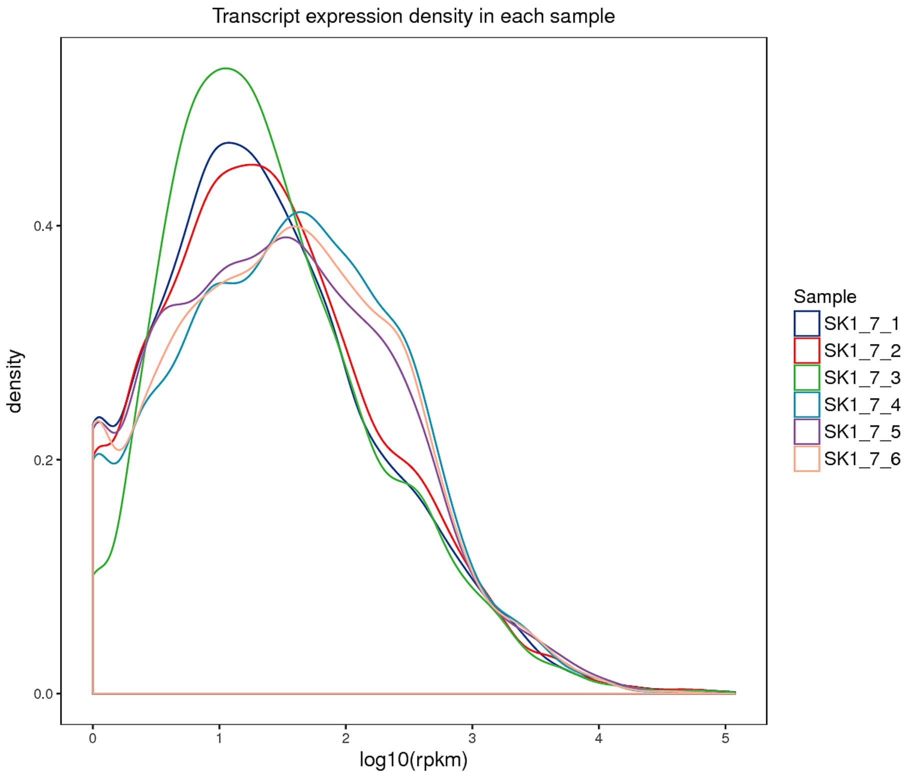

(c)

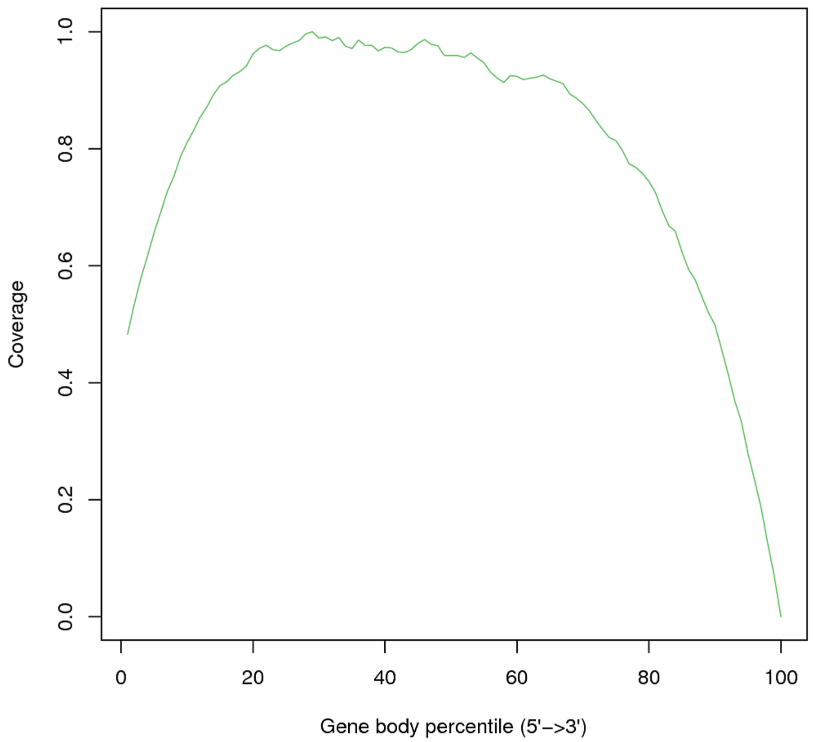

Fig. S2. Histogram (a) and volcano plot (b) of DEGs in SK1-7 grown in FM (Feldspar) medium versus FM (K_2HPO_4) medium.

(a)

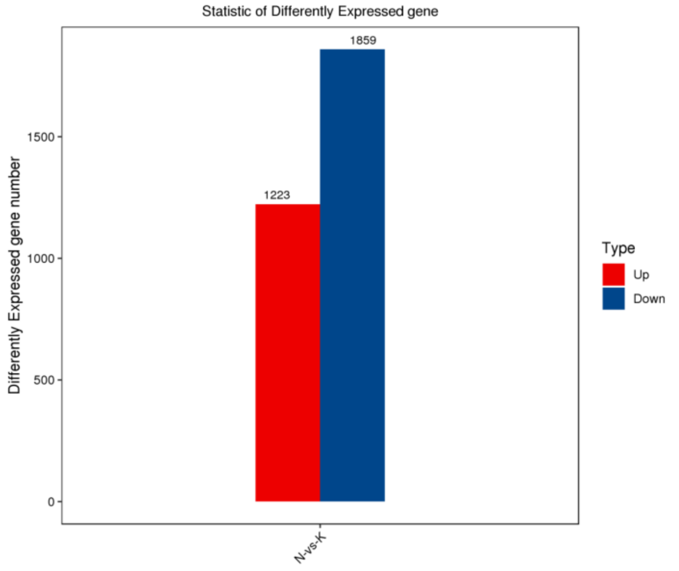

(b)

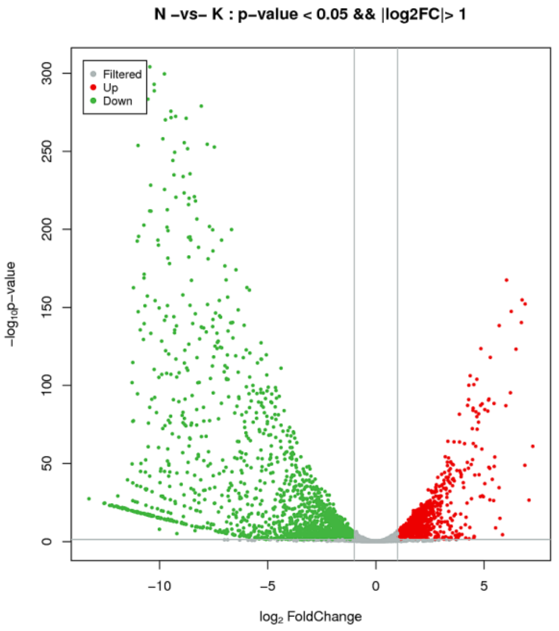

Fig. S3. Directed acyclic graphs of the enriched GO terms for the DEGs of SK1-7. The circles and rectangles are GO terms for the (a) biological process, (b) cellular component, and (c) molecular function categories and their corresponding *P* values. The top 10 enriched GO terms are shown in the rectangles. The darker the circles or rectangles are, the higher the extent of enrichment that occurs in the group.

(a)

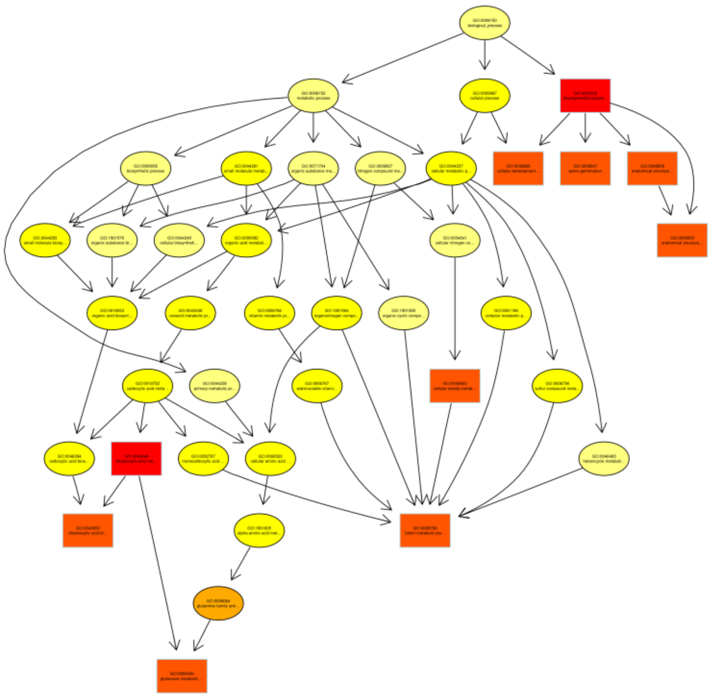

(b)

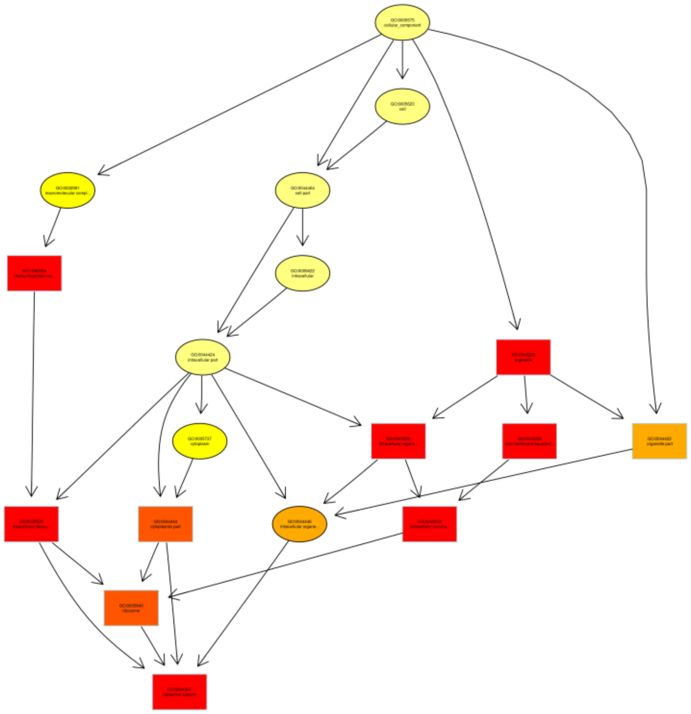

(c)

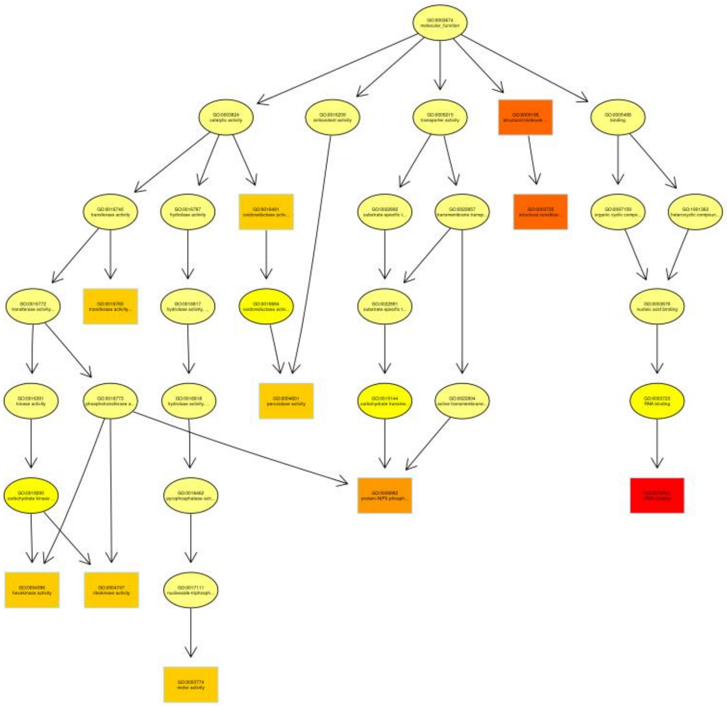

Supplement: Supplemental file 1 — Supplemental material. Download aem.02034-22-s0001.pdf, PDF file, 0.5 MB [file aem.02034-22-s0001.pdf]
